# In silico studies on cytotoxicity and antitumoral activity of acetogenins from *Annona muricata* L

**DOI:** 10.3389/fchem.2023.1316779

**Published:** 2023-11-29

**Authors:** Houéfa Egidia Fallon Adido, Cristian Kallahan Silva Chagas, Gleison Gonçalves Ferreira, Mírian Letícia Carmo Bastos, Maria Fâni Dolabela

**Affiliations:** ^1^ Faculty of Pharmacy, Federal University of Para, Belem, Brazil; ^2^ Postgraduate Program in Pharmaceutical Sciences, Federal University of Para, Belem, Brazil; ^3^ Postgraduate Program in Biodiversity and Biotechnology (BIONORTE), Federal University of Para, Belem, Brazil

**Keywords:** cancer, pharmacokinetics, caspase 3, toxicology, plants, medicinal

## Abstract

As life expectancy increases, the number of people affected by cancer is increasing. The available drugs still cause several adverse reactions, and it is important to look for less toxic drugs that act on resistant cancers. The present study evaluated the antitumor potential of acetogenins. Through a literature review, 44 acetogenins isolated from *Annona muricata* were selected and subjected to *in silico* studies to predict the physicochemical properties, pharmacokinetics (Preadmet and Admet lab), toxicity (Preadmet and Protox II) and molecular docking in caspase 3 (DockThor). For muricatacin, a literature review was carried out for antitumor activity and cytotoxicity. Only muricatacin met all physicochemical criteria, while all compounds showed high cutaneous and intestinal absorption (HIA), moderate permeability in Madin-Darby canine kidney and Caco2 cells, strongly bound plasma proteins, freely crossed the blood-brain barrier, inhibited CYP2C19, CYP2C9 and CYP3A4 and have an affinity for CYP3A4, being metabolized by it, an undesirable characteristic for antitumor drugs. All compounds were toxic in at least one model, while compound 28 was not carcinogenic in rats and mice. Compounds 13, 14, 15, 16, 17 and 28 were selected for molecular docking into Caspase 3. Docking showed hydrophobic interactions, hydrogen and covalent bonds performed to maintain the stability of caspase 3, and cis-uvariamicin IV stood out more through the energies and chemical bonds of this parameter. The chloroform fraction from the methanolic extract of the seeds showed activity against triple-negative breast cancer, both *in vitro* and *in vivo*, and only muricatacin has studies in which the antitumor activity was evaluated *in vitro* and showed to be very promising. In summary, muricatacin and cis-uvariamicin IV appear to be very promising as antitumors, especially cis-uvariamicin IV.

## 1 Introduction

Cancer or malignant neoplasm is a physiological condition, characterized by the disordered growth of cells that tend to invade tissues and organs, compromising their structure and, consequently, their functioning. The causes are multifactorial, usually associated with hormones, immunological conditions, genetics, exposure to chemical substances, viruses, lifestyle habits, among others (Deminice, 2022).

It is estimated that for every three people, one will be diagnosed with cancer, mainly associated with socioeconomic conditions. In 2018, studies showed the occurrence of 18 million new cases of cancer worldwide, and 9.6 million deaths (WHO, 2009). In Brazil, 704 thousand new cases of cancer are expected for each year of the 2023–2025 triennium (INCA, 2022).

In general, cancer is treated through surgery, chemotherapy, radiotherapy, immunotherapy and hormonal therapy, with chemotherapy and radiotherapy being the most requested (Deminice, 2022). Despite advances in treatment, the mortality rate is still high and may be related to late diagnosis, as well as inadequate response to treatment (Oliveira, 2002; Mansoori et al., 2017).

Tumor cells often acquire resistance to chemotherapy drugs, which may involve different mechanisms, in which the induction of multidrug resistance (MDR) is an important one (Nantajit et al., 2015). In addition to tumor resistance, available drugs are still quite toxic and can cause low-severity adverse effects such as fatigue, generalized pain (Pearce et al., 2017), nausea, vomiting (Lorusso et al., 2017) and other gastrointestinal disorders (Pearce et al., 2017), as well as highly serious events such as neurotoxicity and cardiotoxicity (Adão et al., 2013; Simão et al., 2019).

Even with the availability of antitumor drugs, it is necessary to search for therapeutic alternatives that are active in resistant tumors and less toxic to patients. Plants are important sources of pharmaceuticals (Amanda et al., 2022), and the *Annona muricata* is a species that is widely exploited by the cosmetic industry in the manufacture of moisturizers, shampoo, soap, and in the food industry for the preparation of sweets, drinks, ice cream, among others (Maul et al., 2000; De and Abreu, 2019).

The *A. muricata* is also used for medicinal purposes. The bath with leaves is used to treat skin diseases and pain in South Pacific countries, Mauritius and New Guinea (Longuefosse and Nossin, 1996; Sreekeesoon and Mahomoodally, 2014). A decoction of the leaves for ingestion is used as an analgesic in Brazil (Ross, 2010), Martinique (Longuefosse and Nossin, 1996), Mexico and Nicaragua (Ross, 2010).

In Benin (Kossouoh et al., 2007), Caribbean (Joyeux et al., 1995), Cuba (Beyra et al., 2004) and Mexico (Haiat et al., 2009) *A. muricata* is used to control free radicals and strengthening the immune system cells in order to avoid possible discomfort associated with colds, flu and asthma. The natives of Malaysia used the leaves to treat external and internal skin parasites (Badrie and Schauss, 2010). There are still reports of its use to treat malaria in Cameroon, Togo and Vietnam (Nguyen-pouplin and Julie, 2007; Ross, 2010; Boyom and Fekam, 2011). Furthermore, studies have shown this plant could be used to prevent breast, colorectal, lung and prostate cancer (Martínez, 1991; Moghadamtousi et al., 2015; Alonso-Castro and Josabad, 2011; Atawodi Eneojo, 2011; Bussmann and Rainer, 2010, Monigatti et al., 2013).

Chemical studies carried out on *A. muricata* led to the isolation of alkaloids, acetogenins, flavonoids, phenols, cyclopeptides (Moghadamtousi et al., 2015; Coria-téllez and Ana, 2018; Ferreira, 2022), carotenoids, and amides (Vijayameena et al., 2013). The acetogenins were characterized as majority compounds on this species (Mutakin et al., 2022).

Acetogenins, or polyketides, are metabolites derived from fatty acids. They have a long aliphatic chain of 35–38 carbons linked to a g-lactone ring, terminally substituted by β-unsaturated methyl, with tetrahydrofurans (THF) located along the hydrocarbon chain (Mutakin et al., 2022), being the presence of the hydroxyl groups, THF ring and the α, β-unsaturated y-lactone subunit responsible for producing the toxic effect of these compounds (Ferreira, 2022).

Studies have demonstrated that acetogenins have antitumor activity (Syed and Syed, 2016; Machado, 2019) and the mechanisms related to this activity are: 1-inhibition of ubiquinone oxidoreductase NADH, an enzyme essential to complex I of the electron transport chain (Carmen Zafra-Polo et al., 1998; Nakanishi, 2011); 2- inhibition of NADH oxidases in the plasma membrane, an enzyme involved in the production of adenosine triphosphate (ATP) under anaerobic conditions in order to restore coenzyme nicotinamide adenine dinucleotide - NAD levels (Alali and Feras, 1998; Moraes and Vieira, 2016); 3- phosphorylation of histone 3 (H3) through deregulation of the expression of aurora B protein and mitogen-activated protein kinase (pMSK1), which is overexpressed, decreasing phosphorylation at the H3S10 and H3S28 H3 positions; causing consequent cell cycle arrest in the G1 phase; 4- apoptosis activation through the intrinsic (through the mitochondria) and extrinsic (cell death ligands) pathways, activating caspases 8 and 9 (Lee et al., 2011; Gaviria et al., 2018).

Related to toxicity studies, both the ethanolic extract and the dichloromethane fraction from *A. muricata* seeds showed genotoxic activity against meristematic cells of *Alluim cepa* onion, the ethanolic extract was the most cytotoxic; in addition, they also caused a toxic effect against microcrustaceans. (Ferreira, 2022).

Aiming to understand the antitumor potential of different acetogenins isolated from *A. Muricata*, an *in silico* study was carried out to evaluate the physicochemical, pharmacokinetic and toxicological aspects, as well as molecular docking in caspase 3. Furthermore, the most promising acetogenins were selected and investigated whether there are studies on its antitumor activity.

## 2 Methodology

### 2.1 In silico evaluation

#### 2.1.1 Selection of molecules

The molecules were selected based on a bibliographical review, according to some eligibility criteria: having been isolated from *A. muricata*, being classified as acetogenin, having its 2D structure and SMILE code available in the Pubchem database, in addition to responding to at least 3 criteria of Lipinski’s rules of five where molecular mass ≥ 500 Da, LogP ≤ 5; number of hydrogen bond acceptor groups ≤ 10; number of hydrogen bond donor groups ≤ 5 and topological polar surface area (TPSA) ≤ 140 Å. Then, the selected molecules were designed using the program *Marvin Js online*.

#### 2.1.2 Prediction of physicochemical properties

To predict physicochemical properties, the online tool Mcule property calculator was used, considering Lipinski’s rule of five. Calculations of aspects related to the absorption and permeability of cell membranes (partition coefficient, lipophilicity; hydrophilicity) were made using the Molinspiration Online Property Calculation Toolkit website (www.molinspiration.com; Lipinski et al., 2001). In this way, the prediction of bioavailability was carried out considering the octanol-water partition coefficient (miLog P) less than or equal to 5; number of hydrogen bond acceptor groups (nHBA) less than or equal to 10; number of hydrogen bond donor groups (nHBD) less than or equal to 5; molecular mass (MM) less than or equal to 500 Da and the topological polar surface area (TPSA) less than or equal to 140 Å (Lipinski, 2004). For analysis, molecules that did not meet these criteria had low bioavailability (Silva, 2015).

#### 2.1.3 Pharmacokinetics and toxicity

For pharmacokinetic and toxicity predictions, the PreADMET program (version 2.0, Copyright ^©^ 2005–2017) was used, which considers pharmacokinetic properties (A–absorption; D–Distribution; M–Metabolism/Biotransformation; E–Excretion) and evaluation of toxicity parameters (T–Toxicity; Preadmet, 2020).

The pharmacokinetic properties evaluated were: permeability, intestinal absorption, binding to plasma proteins, passage through the blood-brain barrier (BBB) and phase I and II metabolism through induction and inhibition of microsomal cytochrome P-450 (CYP–P450) enzymes, being considered the following parameters: Madin-Darby canine kidney (MDCK) and human colon carcinoma cell line-(Caco-2) (low permeability: <4 nm/s; medium permeability: 4–70 nm/s; high permeability: >70 nm/s; Yazdanian et al., 1998), skin permeability (high: <0.1, low: >0.1); Human Intestinal Absorption - HIA (poorly absorbed: 0%–20%; moderately absorbed: 20%–70%; well absorbed: 70%–100%; Yee, 1997), binding to plasma proteins (weak binding <90%; strong binding >90%) and passage through the BBB (crosses freely: >2; crosses moderately: 2.0–0.1; does not cross: <0.1; Ajay et al., 1999). In the analysis of results for phase I and II metabolism, inhibitory substances for 2 or more CYP enzymes and an inducing substance for at least 1 CYP enzyme were considered.

The toxicity analysis was carried out on *Algae* (Costa et al., 2008), *Daphnia*–through the Daphnia Test (Guilhermino et al., 2000) and Medaka and Minnow fish (Zuncker, 1985) according to the toxicity criteria (Table 1).

**TABLE 1:** Toxicity criteria adopted for the study.

Test	Interval	Classification
Algae test	<1 mg/L	Toxic
	>1 mg/L	Non-toxic
Daphnia test	<0.22 μg/mL	Toxic
	>0.22 μg/mL	Non-toxic
Medaka Minnow fish	<1 mg/L	Very toxic
	1–10 mg/L	Toxic
	10–100 mg/L	Harmful
	>100 mg/L	Extremely toxic

The mutagenicity risk was assessed by the Ames test with the following strains of *Salmonella Typhimurium:* TA100-10RLI and TA 100-NA mutation in His G46e plasmid pKM101 without S9; TA1535- 10RLI and TA1535-NA mutation in His G46 (Ames et al., 1975). The carcinogenic potential of the compounds was evaluated in rats and mice and referred to as: (+) carcinogenic and (–) non-carcinogenic.

To predict acute oral toxicity (oral LD_50_), the online software PROTOX II was used, considering the classification from I to VI (Table 2), according to ABNT NBR 14725–2, 2009 and as provided in Section 1 of the Annex IV of RDC N^o^. 294, 2019.

**TABLE 2:** Parameters adopted for classification of acute oral toxicity (oral LD_50_).

Categories	LD50 (mg/kg)	Assessment
I	0 < DL50 ≤ 5	Extremely Toxic
II	5 < DL50 ≤ 50	Highly Toxic
III	50 < DL50 ≤ 300	Moderately Toxic
IV	300 < DL50 ≤ 2 000	Little Toxic
V	2 000 < DL50 ≤ 5,000	Unlikely to Cause Acute Damage
VI	DL50 > 5,000	No damage

LD_50_ = median lethal dose.

### 2.2 Molecular docking

For this trial, the effector Caspase 3 (cysteine-aspartic protease) receptors were the therapeutic targets for the action of acetogenins. This caspase was chosen due to its importance in the process of apoptosis, programmed cell death. This enzyme is a key zymogen in cellular apoptosis and is not activated until it is cleaved by initiator caspases during apoptotic flux.

To perform molecular docking, the caspase 3 structure was downloaded in two dimensions (2D) from the RCSB Protein Data Bank PDB website: 1 nms. Just as the 2D structures of acetogenins were downloaded in PDB format using the chemSketch program, Docking was carried out.

Molecular docking was carried out using the DockThor program to verify whether the investigated molecules had affinity for caspase 3 receptors, possible therapeutic targets. Docking was performed out using the grid center of the irreversible inhibitor already co-crystallographed with Caspase, to identify whether acetogenins bind to the same site. (Almeida, 2011) For this, some parameters were considered: the addition of hydrogen to the ligand, grid center: X = -9.091719; Y = -4.028625; Z = 24.242812; and the grid size: X:20Å, Y:20Å and Z:20 Å. The search was done by the standard algorithm with 1,000,000 reviews, population of 750 in 24 runs. This process was repeated for each acetogenin.

The results were observed using the Biovia Discovery studio visualizer 4.5 software, a program for visualizing the structure of proteins and small molecules and analyzing data. (Biovia Systemes, 2015).

### 2.3 Literature review

A bibliographic survey was carried out to correlate the results obtained and information in the literature on the antitumor activity of acetogenins, as well as the cell lines involved in this activity, for that, the following databases were searched: Google Scholar, Pubmed, Science direct, CAPES journal portal and SciELO.

## 3 Results and discussion

**FIGURE 1:**
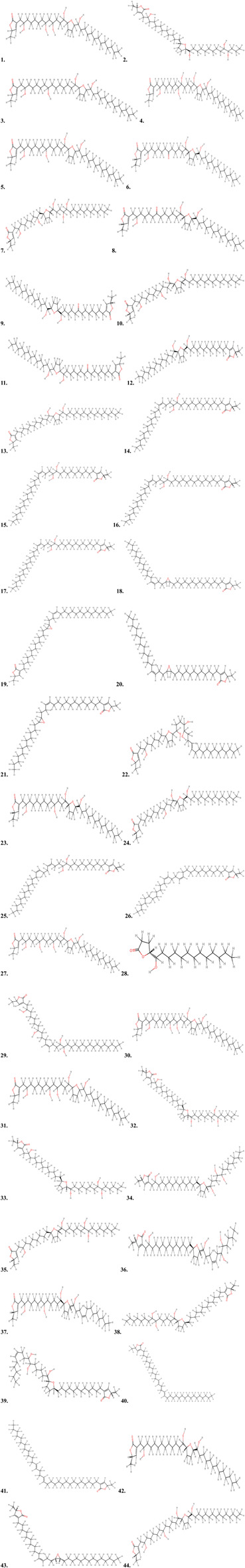
Acetogenins isolated from *A. muricata*.

Several chemical studies on *A. muricata* have reported the isolation of acetogenins, with more than 100 different acetogenins isolated (Moghadamtousi et al., 2015). Because not all acetogenins have a 2D structure and SMILE code available in the Pubchem database, they were excluded from the present study, wherefore only 44 acetogenins were selected, whose structures and names are shown in Figure 1:

1-Muricatocin B; 2-annocatalin; 3- annomontacin; 4-annomuricin A; 5-annonacin A; 6- annonacinone; 7- annopentocin A; 8-annoreticuin-9-one; 9-cis-corossolone; 10-cis-goniothalamicin; 11-cis-reticulatacin-10-one; 12-cis-solamin; 13-cis-uvariamicin IV; 14- cohibin A; 15-cohibin B; 16- cohibin C; 17-cohibin D; 18-epomusenin-A; 19-epomusenin-B; 20-epoxymurin A; 21- epoxymurin B; 22-gigantetronenin; 23- javoricin; 24-longifolicin; 25-montecristin; 26- muricadienin; 27-muricapentocin; 28-muricatacin; 29-muricatenol; 30-muricatocin A; 31-muricatocin B; 32-muricin A; 33-muricin B; 34-muricin C; 35- muricin E; 36-muricin F; 37- muricin G; 38- muricin H; 39- muricin I; 40-muridienin 3; 41-muridienin 4; 42-murisolin; 43-sabadelin; 44- xylomaticin.

### 3.1 In silico studies of acetogenins

Regarding physicochemical properties, from 44 compounds, only 28 met all the criteria of Lipinski’s rule of five. The remaining compounds violated two criteria established by this rule: log *p* > 5 and molecular mass>500 Da (Table 3). Although these molecules violated the rule, it does not mean that their therapeutic potential should be ignored (Lipinski, 2004; Chagas and Kallahan, 2022).

**TABLE 3:** Values prediction ​​of physicochemical properties of acetogenins isolated from *Annona muricata* using *Mcule* property calculator.

Compounds	MM	LogP	TPSA	nHBA	nHBD
1	612.8766	6.0321	136.6800	8	5
2	596.8772	7.0613	116.4500	7	4
3	624.9303	7.8415	116.4500	7	4
4	612.8766	6.0321	136.6800	8	5
5	596.8772	7.0613	116.4500	7	4
6	596.8613	7.2695	113.2900	7	3
7	612.8766	6.0321	136.6800	8	5
8	594.8613	7.2695	113.2900	7	3
9	594.8613	7.2695	113.2900	7	3
10	596.8772	7.0613	116.4500	7	4
11	606.9150	9.0789	93.0600	6	2
12	564.8784	9.1197	75.9900	5	2
13	592.9315	9.8999	75.9900	5	2
14	548.8790	9.9085	66.7600	4	2
15	548.8790	9.9085	66.7600	4	2
16	576.9321	10.6887	66.7600	4	2
17	576.9321	10.6887	66.7600	4	2
18	558.9168	11.7343	38.8300	3	0
19	558.9168	11.7343	38.8300	3	0
20	530.8637	10.9541	38.8300	3	0
21	530.8637	10.9541	38.8300	3	0
22	622.9144	7.6175	116.4500	7	4
23	596.8772	7.0613	116.4500	7	4
24	580.8778	8.0905	96.2200	6	3
25	574.9162	10.4647	66.7600	4	2
26	514.8643	11.7429	26.3000	2	0
27	612.8766	6.0321	136.6800	8	5
28	284.4336	4.3639	46.5300	3	1
29	608.9309	8.6303	107.2200	6	4
30	612.8766	6.0321	136.6800	8	5
31	612.8766	6.0321	136.6800	8	5
32	596.8772	7.0613	116.4500	7	4
33	596.8772	7.0613	116.4500	7	4
34	596.8772	7.0613	116.4500	7	4
35	568.8241	6.2811	116.4500	7	4
36	594.8613	6.8373	116.4500	7	4
37	594.8613	6.8373	116.4500	7	4
38	580.8778	8.0905	96.2200	6	3
39	606.9150	8.6467	96.2200	6	3
40	542.9174	12.5231	26.3000	2	0
41	542.9174	12.5231	26.3000	2	0
42	580.8778	8.0905	96.2200	6	3
43	530.8637	10.9541	38.8300	3	0
44	624.9303	7.8415	116.4500	7	4

The 43 molecules that have a greater molecular weight MM > 500Da may have difficulty to cross the plasma membrane. Each molecule’s topological polar surface area (TPSA) was less than 140 Å, which is justified by the lower bond between the hydrogen acceptors and donors, respectively ≤10 and ≤5. Therefore, the muricatacin (Figure 1 Compound 28; Table 3) would be the most promising for physicochemical properties, being able to express biological or pharmacological activity and be used orally in humans (Lipinski, 2004).

Lipinski rule: LogP- oil-water partition coefficient ≤5; TPSA: topological polarized surface area ≤140 Å; nHBA: Hydrogen bond acceptors ≤10; nHBD-number of hydrogen bond donor groups ≤5; MM-molecular mass≤ 500 g/mol (LIPINSKI, 2004).

In the pharmacokinetic prediction, all molecules presented high skin absorption, (Table 4), acetogenins have a lipophilic character (Barata et al., 2009), and can be used in dermocosmetics. However, permeabilities in MDCK (Madin-Darby canine kidney) cells and Caco2 (human colon carcinoma epithelial cells) were moderate, except for molecule 28 (Table 4). When assessing permeability in MDCK (Madin-Darby canine kidney) cells, the elimination rate of each molecule from the body was measured (Miranda et al., 2022). Molecule 28 showed high MDCK permeability (Table 4), maybe due to its lower molecular mass (Chen and Eugene, 2018). All molecules appear to have moderate permeability for Caco2 cells (Table 4), suggesting that the rate of intestinal absorption is moderate (Miranda et al., 2022) through the passive diffusion mechanism (Marinho and Thaisa, 2017; Chen and Eugene, 2018), which may be related to its high molecular mass. However, the studied acetogenins appear to have high absorption in the small intestine by human intestinal absorption analysis (Table 4). In a chemotherapy treatment, the oral route offers the benefits of convenience, the possibility of taking the medication at home without the need to go to clinics and hospitals (Vasconcelos et al., 2022). Also, acetogenins have an acidic character (Barata et al., 2009) and must have albumin affinity (Horta and Silvania Sofia dias, 2018).

**TABLE 4:** Prediction of pharmacokinetic properties of acetogenins isolated from *Annona muricata* using *Preadmet*.

Compounds	Absorption				Distribution		Metabolism	
	Cutaneous	MDCK	Caco 2	HIA	PP	BHE	CPY inhibition	CYP phase 1
1	H	L	M	H	S	F	CYP2C19, 2C9,3A4	3A4 FR
2	H	M	M	H	S	F	CYP2C19, 2C9,3A4	3A4 FR
3	H	M	M	H	S	F	CYP2C19, 2C9,3A4	3A4 FR
4	H	M	M	H	S	F	CYP2C19, 2C9,3A4	3A4 FR
5	H	M	M	H	S	F	CYP2C19, 2C9,3A4	3A4 FR
6	H	M	M	H	S	F	CYP2C19, 2C9,3A4	3A4
7	H	L	M	H	S	F	CYP2C19, 2C9,3A4	3A4 FR
8	H	M	M	H	S	F	CYP2C19, 2C9,3A4	3A4
9	H	M	M	H	S	F	CYP2C19, 2C9,3A4	3A4
10	H	M	M	H	S	F	CYP2C19, 2C9,3A4	3A4 FR
11	H	M	M	H	S	F	CYP2C19, 2C9,3A4	3A4
12	H	M	M	H	S	F	CYP2C19, 2C9,3A4	3A4 FR
13	H	M	M	H	S	F	CYP2C19, 2C9,3A4	3A4 FR
14	H	M	M	H	S	F	CYP2C19, 2C9,3A4	3A4 FR
15	H	M	M	H	S	F	CYP2C19, 2C9,3A4	3A4 FR
16	H	M	M	H	S	F	CYP2C19, 2C9,3A4	3A4 FR
17	H	M	M	H	S	F	CYP2C19, 2C9,3A4	3A4 FR
18	H	M	M	H	S	F	CYP2C19, 2C9,3A4	3A4 FR
19	H	M	M	H	S	F	CYP2C19, 2C9,3A4	3A4 FR
20	H	M	M	H	S	F	CYP2C19, 2C9,3A4	3A4 FR
21	H	M	M	H	S	F	CYP2C19, 2C9,3A4	3A4 FR
22	H	M	M	H	S	F	CYP2C19, 2C9,3A4	3A4 FR
23	H	M	M	H	S	F	CYP2C19, 2C9,3A4	3A4 FR
24	H	M	M	H	S	F	CYP2C19, 2C9,3A4	3A4 FR
25	H	M	M	H	S	F	CYP2C19, 2C9,3A4	3A4 FR
26	H	M	M	H	S	F	CYP2C19, 2C9,3A4	NO
27	H	M	M	H	S	F	CYP2C19, 2C9,3A4	3A4
28	H	M	M	H	S	F	CYP2C19, 2C9,3A4	NO
29	H	M	M	H	S	F	CYP2C19, 2C9,3A4	3A4 FR
30	H	M	M	H	S	F	CYP2C19, 2C9,3A4	3A4 FR
31	H	L	M	H	S	F	CYP2C19, 2C9,3A4	3A4 FR
32	H	M	M	H	S	F	CYP2C19, 2C9,3A4	3A4 FR
33	H	M	M	H	S	F	CYP2C19, 2C9,3A4	3A4 FR
34	H	M	M	H	S	F	CYP2C19, 2C9,3A4	3A4 FR
35	H	M	M	H	S	F	CYP2C19, 2C9,3A4	3A4 FR
36	H	M	M	H	S	F	CYP2C19, 2C9,3A4	3A4 FR
37	H	M	M	H	S	F	CYP2C19, 2C9,3A4	3A4 FR
38	H	M	M	H	S	F	CYP2C19, 2C9,3A4	3A4 FR
39	H	M	M	H	S	F	CYP2C19, 2C9,3A4	3A4 FR
40	H	M	M	H	S	F	CYP2C19, 2C9,3A4	NO
41	H	M	M	H	S	F	CYP2C19, 2C9,3A4	NO
42	H	M	M	H	S	F	CYP2C19, 2C9,3A4	3A4 FR
43	H	M	M	H	S	F	CYP2C19, 2C9,3A4	3A4 FR
44	H	M	M	H	S	F	CYP2C19, 2C9,3A4	3A4 FR

Despite the high molecular mass of compounds, the results suggest they cross the blood-brain barrier (BBB) freely (Table 4), probably due to their high lipid solubility (Chagas and Kallahan, 2022) (Table 3). It is noteworthy that different types of tumors develop in the central nervous system (CNS) and their growth can be rapid, making it important to develop drugs that can be administered orally and reach therapeutic concentrations in the CNS.

Another common point among acetogenins is the inhibitory potential of CYP2C19, 2C9 and 3A4, important in drug metabolism, mainly cyp3a4, which is the most abundant and its inhibition will be unfavorable for an antitumor drug. (Sukprasong, et al., 2021). The ability to inhibit CYP may be related to physicochemical properties as the greater the number of hydrogen acceptors, the greater the inhibitory potential. Furthermore, by inhibiting CYP 3A4 and others, the compound can interfere with the metabolism of different classes of drugs, requiring dose adjustment (De Lima and Cyntia, 2018). All are weakly metabolized by CYP3A4, and compounds 6, 8, 9, 11 and 27 also undergoing phase 1 metabolism (Table 4), giving rise to more polar metabolites, which are more easily excreted via the kidneys (Dolabela and Fani, 2018).

MDCK: Madin-Darby canine kidney; Caco2: Human Colon Adenocarcinoma Cells; PP-plasma protein; BBB: blood brain barrier; CYP: citrochrome P450; HIA: human intestinal absorption, S*: strongly; F *: freely; NO: not observed; W - weakly; H - high; L: low; M-medium.

In addition to the physicochemical and pharmacokinetic aspects, the toxic potential of acetogenins was evaluated using different models (Table 5). Only acetogenins 13 to 21, 25, 26 and 29 were not toxic to algae (Table 5), this model was used to predict acute oral toxicity in relation to death (Guilhermino et al., 2000). The same non-toxic compounds for algae were not toxic for Daphnia (Table 5), which can be used to predict acute and subchronic toxicities (Venturi, 2018).

**TABLE 5:** Toxicity of acetogenins isolated from *Annona muricata.*

Compounds	Algae test	Daphnia test	Medaka fish	Minnow fish	Ames	Carcino rat/mice *
1	T	T	T	T	N	N/P
2	T	T	T	T	N	N/P
3	T	T	T	T	N	N/P
4	T	T	T	T	N	N/P
5	T	T	T	T	N	N/P
6	T	T	T	T	N	N/P
7	T	T	T	T	N	N/P
8	T	T	T	T	N	N/P
9	T	T	T	T	N	P/P
10	T	T	T	T	N	N/P
11	T	T	T	T	N	P/P
12	T	NT	T	T	N	P/P
13	NT	NT	T	T	N	N/P
14	NT	NT	T	T	N	N/P
15	NT	NT	T	T	N	N/P
16	NT	NT	T	T	N	N/P
17	NT	NT	T	T	N	N/P
18	NT	N	T	T	N	P/P
19	NT	NT	T	T	N	P/P
20	NT	NT	T	T	N	P/P
21	NT	NT	T	T	N	P/P
22	T	T	T	T	N	N/P
23	T	T	T	T	N	N/P
24	T	T	T	T	N	N/P
25	NT	NT	T	T	N	P/P
26	NT	NT	T	T	N	P/P
27	T	T	T	T	N	N/P
28	T	T	VT	VT	TA100-10RLI	N/N
29	NT	NT	T	T	N	N/P
30	T	T	T	T	N	N/P
31	T	T	T	T	N	N/P
32	T	T	T	T	N	N/P
33	T	T	T	T	N	N/P
34	T	T	T	T	N	N/P
35	T	T	T	T	N	N/P
36	T	T	T	T	N	N/P
37	T	T	T	T	N	N/P
38	T	T	T	T	N	N/P
39	T	T	T	T	TA100-10RLI	N/P
40	NT	NT	T	T	N	P/P
41	NT	NT	T	T	N	P/P
42	T	T	T	T	N	N/P
43	NT	NT	T	T	N	P/P
44	T	T	T	T	N	N/P

T: toxic; NT: non-toxic; VT:very toxic N: negative; P: positive. Parameters: Algae - < 1 mg/L toxic; >1 mg/L non-toxic (Costa, et al., 2008); Daphnia Test: <0.22 μg/mL toxic; >0.22 μg/mL - non-toxic (Guilhermino, et al., 2000); Test on Medaka and Minnow fish: <1 mg/L - very toxic; 1–10 mg/L-toxic; 10–100 mg/L-harmful and >100 mg/L-extremely toxic (Zuncker, 1985), Carcino Rat/mice* = carcinogenicity in rat/mice. T-toxic, NT-non-toxic, VT-very toxic, N-negative, P-positive.

All compounds were toxic to fish (Table 5), suggesting acute and subchronic toxicity, as well as changes in different organs (Bauer, 2017). Through the results obtained in Algae, Daphnia and fish, we suggest the compounds with the greatest acute and subchronic toxic potential are: 13, 14, 15, 16, 17, 18, 19, 20, 21, 25, 26 and 29. However, it is important to consider the mutagenic and carcinogenic potential. Except for compound 28, all compounds showed carcinogenic potential for mice and compounds 11, 12, 18 to 21, 25, 26, 40, 41, 43 showed carcinogenic potential in rats (Table 5).

Only compounds 28 and 39 showed mutagenic potential for *Salmonella typhimurium* (TA 100-10RLI; Table 5), this strain is sensitive for the detection of mutagenicity and contain a pKM101 plasmid and mutation in His G46 (Dayse et al., 2020). Analyzing the toxicity results (Table 5), we suggest the compounds with the highest toxic potential are: 18, 21, 25 and 26.

In acute oral toxicity, only the molecule 28 (muricatacin) presented LD_50_ > 5,000, being classified in category VI, it appears to cause no harm and does not present immunotoxicity or cytotoxic effects. Molecules 9,11,12,13,24,26,38,40 and 41 appear unlikely to cause harm, but all have immunotoxic or genotoxic potential. On the other hand, molecules 7, 22, 36 and 37 appear to be extremely toxic, presenting cytotoxic and immunotoxic potential (Table 6). Based on these results, we can suggest that the least promising molecules are: 7, 18, 21, 22, 25, 26, 36 and 37 (Tables 5, Table 6).

**TABLE 6:** Acute oral toxicity values ​​(oral LD_50_) of acetogenins isolated from *Annona muricata* using PROTOX II online software.

Compounds	LD_50_ (mg/kg)	Toxicity class	Side effects
1	400	IV	I/C
2	400	IV	I/C
3	400	IV	I/C
4	400	IV	I/C
5	347	IV	I/C
6	400	IV	I/C
7	46	II	I/C
8	400	IV	I/C
9	5,000	V	I/C
10	400	IV	I/C
11	5,000	V	I/M
12	5,000	V	I/C
13	5,000	V	I/C
14	841	IV	I/C
15	841	IV	I/C
16	841	IV	I/C
17	841	IV	I/C
18	900	IV	I
19	900	IV	I
20	900	IV	I
21	900	IV	I
22	46	II	I/C
23	400	IV	I/C
24	5,000	V	I/C
25	841	IV	I/C
26	5,000	V	I
27	400	IV	I/C
28	26,000	VI	NO
29	100	III	I/C
30	400	IV	I/C
31	400	IV	I/C
32	400	IV	I/C
33	400	IV	I/C
34	400	IV	I/C
35	400	IV	I/C
36	46	II	I/C
37	46	II	I/C
38	5,000	V	I/C
39	841	IV	I/C
40	5,000	V	I
41	5,000	V	I
42	400	IV	I/C
43	900	IV	I
44	400	IV	I/C

LD_50_-lethal dose 50%. NO = nothing observed. I-immunotoxicity, C-cytotoxicity, M-mutagenicity. Category I: 1 < LD_50_≤ 5 mg/kg - Extremely Toxic; Category II: 5 < LD_50_ ≤ 50 mg/kg- Highly Toxic; Category III: 50 < LD_50_ ≤ 300 mg/kg - Moderately Toxic; Category IV: 300 < LD_50_ ≤ 2,000 mg/kg - Low Toxic; Category V: 2000 < LD_50_ ≤ 5,000 Unlikely to Cause Acute Damage; Category VI: DL_50_ > 5,000 No damage.

Source: ABNT NBR, 2009; RDC No. 294, 2019.

### 3.2 Molecular docking simulation

To select the molecules for docking, physicochemical and pharmacokinetic aspects were considered, in these criteria the most promising was molecule 28 (Tables 3, Table 4). Furthermore, the results in toxicity prediction studies were evaluated, in which some molecules were not toxic to Algae, Daphnia, nor mutagenic nor carcinogenic to rats (13, 14, 15, 16 and 17). Regarding acute oral toxicity, these compounds appear to be low toxic (14, 15, 16 and 17) or unlikely to cause harm (13). In summary, the most promising molecule is 28, but the other molecules were included for the next stage of the study (13–17).

**FIGURE 2:**
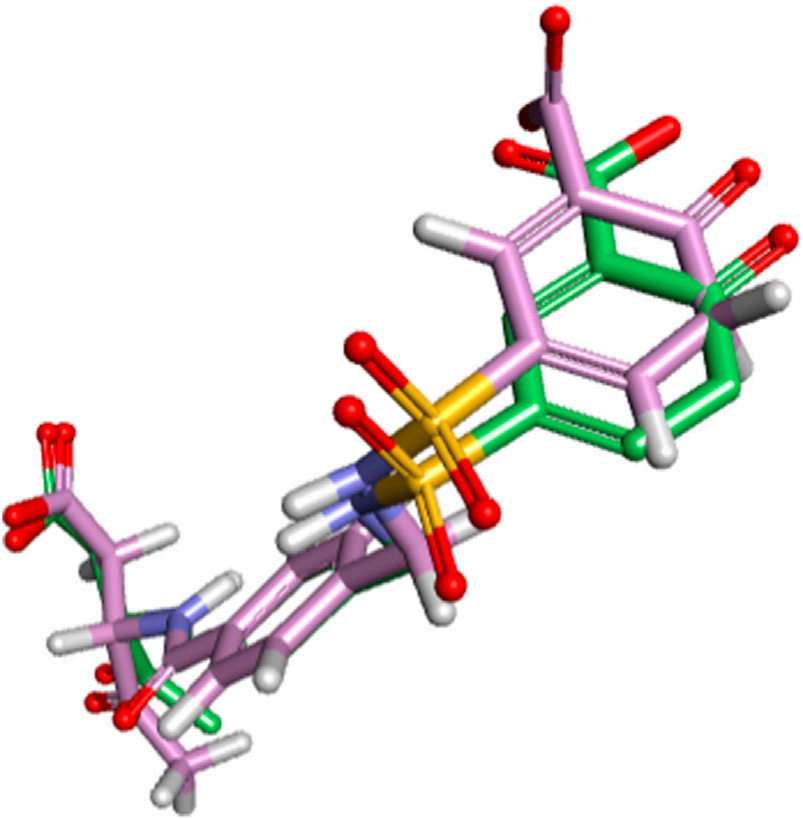
Structure obtained by redocking for the co-crystallographed ligand (irreversible inhibitor) with 1mns target (caspase 3).

For validating the docking protocol applied in the next stage, redocking was carried out, it showed that the best fit of the co-crystallized ligand would be at the value of Root Mean Square deviation - RMSD = 2.09Å (Figure 2), and according to the literature, the prediction of the binding mode using docking must present an RMSD value < 2.0 Å, when superimposed on the crystallographic pose of the ligand (Ramos and Ryan, 2019; Araújo and Pedro, 2020; Mascarenhas and Mercia, 2021). However, the reference values accepted in the literature are RMSD up to 2 Å for rigid structures and up to 2.5 Å for flexible structures (Xiao et al., 2018; Santos et al., 2020; Dardenne, 2021; Altê Alves, 2022). Therefore, this result shows the model was suitable for predicting the docking profile between the ligand molecule and the active site of the selected target.

For the formation of the enzyme complex with each acetogenin, cis-uvariamicin IV; cohibin A; cohibin B; cohibin C; cohibin D; muricatacin, the binding free energies were respectively -8.436; -9.151; -9.339; -8.715; -9.275 and -7.632 kcal/mol. The results demonstrated ligands are capable of interacting with the enzyme in a favorable way and cohibin B would have the highest affinity with a minimum score of -9.339 (kcal/mol). As for total energy, it was higher with compound 13 (-48,007 kcal/mol) and lower for 28 (-6,123 kcal/mol). Van der Waals energy was higher for molecule 14 (-30,613 kcal/mol) and lower for 28 (-15,609 kcal/mol). The interaction and electrostatistic energies were respectively higher for the numbers 17 (-15.830 kcal/mol) and 13 (-23.064 kcal/mol) and lower for the compounds 28 (-27,981 kcal/mol) and 14 (-7,904 kcal/mol). Compound 13 cstood out most with its total and electrostatistic energies, important energy for the formation of chemical bonds (Table 7). Although compound 14 has stronger bonds, such as the pi-alkyl type, they require more energy to form bonds, which justifies their lower value of electrostatic energy, due to the different nature of the bond.

**TABLE 7:** Binding affinity between acetogenins isolated from *Annona muricata* and caspase 3 obtained from molecular docking.

Name	Score	T. Energy	vdW energy	I. Energy	Electrostatic energy
13	−8.436	−48.007	−17.543	−40.607	−23.064
14	−9.151	−33.976	−30.613	−38.517	−7.904
15	−9.339	−6.308	−29.885	−42.618	−12.733
16	−8.715	−32.175	−22.422	−39.001	−16.579
17	−9.275	−42.876	−29.654	−45.484	−15.830
28	−7.632	−6.123	−15.609	−27.981	−12.332
Ligante	−7.925	−31.332	21.954	−91.792	−113.746

T. Energy = total energy; vdW Energy = Van der Waals energy; I. Energy = interaction energy.

**FIGURE 3:**
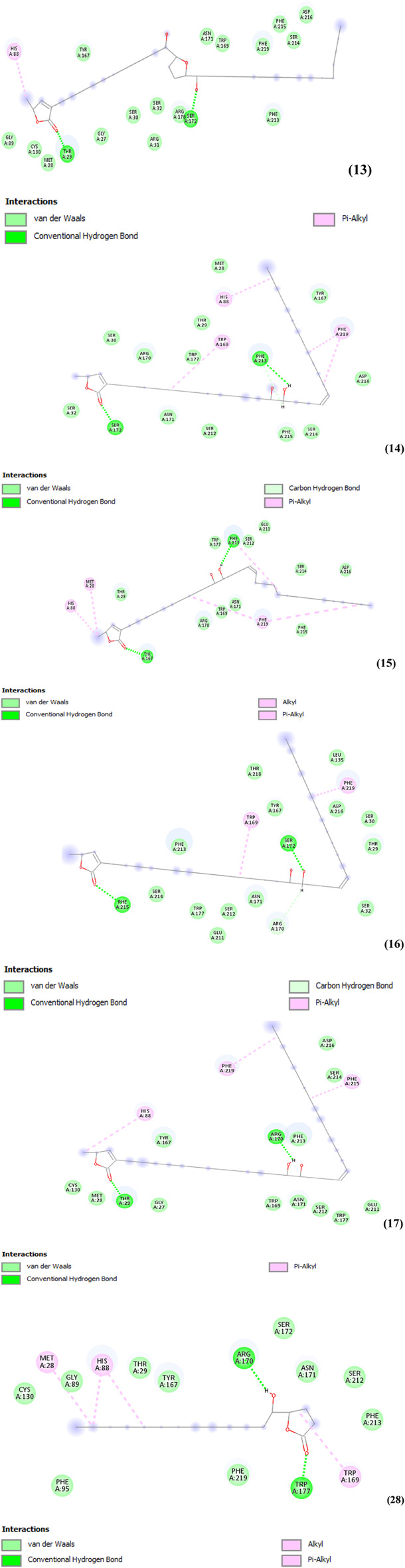
(Continued) Representation of 2D interactions of cis-uvariamicin IV (13), cohibin A (14), cohibin B (15), cohibin C (16), cohibin D (17) and muricatacin (28) in the active site of the caspase 3 enzyme. Image generated with Discovery Studio 4.5 Visualizer.

The results suggest that Cis-uvariamicin IV performs hydrophobic pi-alkyl interaction with HIS 88 and hydrogen bonds on THR 29 and SER 172. Cohibin compounds A, B, C and D perform hydrophobic pi-alkyl bonds with TRP 169, HIS 88, PHE 219, MET 28, PHE 213 and PHE 215; conventional hydrogen bonds with SER172, TYR 167, PHE 213, THR 29, ARG 170 and PHE 215; covalent bonds with PHE 213. Muricatacin makes hydrophobic alkyl bonds in ME28 and pi-alkyl in HIS 88, and TRP 169; conventional hydrogen bonds in ARG 170 and TRP 177 Even though both bonds are hydrophobic, the alkyl-type bond tends to carry out a nucleophilic interaction that favors the interaction between the ligand and the receptor (Figure 3). The distances were relatively smaller between hydrogen bonds (between 1 and 2.5 Å) and covalent bonds (between 2 and 3.5 Å) and larger for hydrophobic bonds (4–5.5 Å).

The approach of hydrophobic surfaces contributes to the destruction of the organized structure of water, with interactions between ligands and receptors due to the increase in entropy associated with the disorganization of the system. Hydrogen bonds are important interactions for maintaining a protein’s structure, and drugs that interact through covalent bonds inactivate the receptor site and consequently the action of enzymes (Barreiro and Fraga, 2015). Table 8 also shows compound 13 establishes four hydrogen bonds, with relatively smaller distances with the amino acids and consequently stronger bonds.

**TABLE 8:** Interactions between ligands and amino acid residues.

Ligands	Amino acid	Interactions	Distances
13	HIS88 THR29 THR29 SER172 SER172	Pi-alkyl L-H L-H L-H L-H	4.79 1.74 1.58 2.03 1.49
14	SER172 SER172 TRP169 HIS88 PHE213 PHE213 PHE219 PHE219	L-H L-H Pi-alkyl Pi-alkyl C-H C-H Pi-alkyl Pi-alkyl	1.79 2.21 5.39 4.12 2.84 3.04 5.44 4.62
15	HIS88 MET28 TYR167 PHE213 PHE213 PHE219 PHE219	Pi-alkyl Pi-alkyl L-H Pi-alkyl L-H Pi-alkyl Pi-alkyl	4.81 4.84 1.79 5.17 1.93 5.48 4.84
16	PHE215 TRP169 SER172 SER172 PHE219	L-H Pi-alkyl L-H L-H Pi-alkyl	1.84 5.13 2.08 1.51 4.85
17	THR29 THR29 HIS88 ARG170 PHE219 PHE215	L-H L-H Pi-alkyl L-H Pi-alkyl Pi-alkyl	1.75 1.81 5.20 1.69 4.50 4.95
28	MET28 HIS88 HIS88 ARG170 TRP177 TRP169	Alkyl Pi-alkyl Pi-alkyl L-H L-H Pi-alkyl	4.72 4.31 5.26 1.76 1.50 5.43

TYR (tyrosine), HIS (histidine), ARG (arginine), PHE (phenylalanine), MET (methionine), THR (threonine), SER (serine), TRP (tryptophan), L-H = hydrogen bond, C-H (covalent bond). cis-uvariamicin IV (13); cohibin A (14); cohibin B (15); cohibin C (16); cohibin D (17) and muricatacin (28).

### 3.3 Antitumor and cytotoxicity activity of muricatacin on literature

The last stage of this study was to correlate our results with studies evaluating the antitumor activity of the selected acetogenins (13–17 and 28), however, we observed the scarcity of *in vitro* and *in vivo* studies. One factor that may limit these studies is the chemical aspects, because the plant may contain several acetogenins and some may be isomers (Sun, et al., 2014), which makes it difficult to isolate compounds.

Another limiting factor may be the low concentrations of acetogenins in the extracts, requiring a large amount of plant material to isolate enough for compounds identification and tests. A very positive point is the storage location of acetogenins in *A. muricata*, the majority are stored in the seeds (3, 5, 6, 8, 10, 17, 22, 23, 24, 28, 29, 32, 33, 34, 35, 36, 37, 38, 39, 42 and 44) (Moghadamtousi, et al., 2015), as the fruit pulp is widely used in the food industry and the seeds discarded, these seeds can be reused and not discarded in the environment.

Despite being very promising molecules, there is still a lack of studies about them, and the available ones were carried out with fractions from extracts containing acetogenins. A study carried out with the chloroform fraction of the methanolic extract of *Annona muricata* seeds (CMAM) showed activity against triple-negative breast cancer (TNBC), both *in vitro* and *in vivo* (Kariyil and Bibu, 2021). We emphasize that TNBC treatment is still a challenge, as it usually does not respond satisfactorily to available therapy (Garrido Castro et al., 2019; So, 2022).

The anticancer effect of CMAM may be related to ROS dependent caspase activated mitochondria-mediated apoptosis and S-phase arrest in TNBCs. Furthermore, the results indicated the presence of annonaceous acetogenins, especially muricatacin, that may be contributing to the anticancer effect of CMAM (Kariyil and Bibu, 2021).

In one study, the MTT (3-(4,5-dimethylthiazol-2yl)-2,5-diphenyl tetrazoline bromide) cell viability assay was applied to evaluate the cytotoxic potential of CMAM in TNBC cells (MDA- MB-231, BT-549 and 4T1) and the normal breast epithelial cells, MCF10A. The results showed the plant fraction was cytotoxic to TNBC cells and the level of damage was concentration dependent. The IC_50_ values were 2.5 ± 0.14, 4.8 ± 0.3 and 4.5 ± 0.16 μg/mL, respectively, 4TI, BT-549 and MDA-MD-231. However, for normal mammary epithelial cell line, MCF10A, CMAM showed lower cytotoxicity with IC_50_ value of 81.1 ± 2.28 μg/mL, demonstrating its differential cytotoxicity towards cancerous and normal cells (Kariyil and Bibu, 2021).

Aiming to evaluate the selectivity of CMAM antitumor activity, the selectivity index (IC_50_ 4/IC_50_ 1, 2 or 3) was established, and the MDA-MB-231 lineage was the most selective with SI = 32 (Table 9).

**TABLE 9:** Cytotoxicity and selectivity of the chloroform fraction of the methanolic extract of Annona muricata seeds in TNBC cell lines.

Lineage/CI_50_ µg/mL ±SD		SI
Tumor	Normal	
1–2.5 ± 0.14 2–4.8 ± 0.3 3–4.5 ± 0.16	4–81.1 ± 2.28	1–32.44 2–16,9 3–18.02

SI-selectivity index; CI-cytotoxicity index; 1-MDA-MB-231, a human epithelial breast cancer adenocarcinoma cell line derived from the metastatic site; 2-4T1, an adherent Balb/cfC3H mouse mammary tumor epithelial cell line; 3-BT-549, an adherent human breast ductal adenocarcinoma cell line; 4-MCF10A, an adherent human normal breast cell line. Source: Kariyil and Bibu, 2021 adapted by the authors.

Despite their high selectivity for cancer cells, some acetogenins have neurotoxic potential, leading to neurodegenerative diseases such as Alzheimer’s and Parkinson’s (Lannuzel et al., 2007; Chuzeville, 2015; Das and Nascimento, 2022). Acetogenins can promote the intracellular aggregation of a tau protein known as tauopathy, leading to the redistribution and accumulation of tau in somatodendrites (Rottscholl, et al., 2016).

*In vitro* and *in vivo* studies indicated anonacin can cause the development of signs associated with tauopathies, such as tau hyperphosphorylation, retrograde mitochondrial transport, tau redistribution from the axon to the neuronal body, and cell death (Lannuzel et al., 2007; Guillopé, et al., 2011; Bruch, et al., 2014; Rottscholl, et al., 2016). Different studies suggest that anonacin (mono-tetrahydrofuran) causes a reduction in ATP in neurons, through the inhibition of mitochondrial complex I (Lannuzel et al., 2003; Champy et al., 2004; Escobar-khondiker et al., 2007; Potts, et al., 2012; Rottscholl et al., 2016).

## 4 Conclusion

The *in silico* study allowed to observe that almost all acetogenins are fat-soluble compounds, cross the blood-brain barrier and inhibit CYP2C19, CYP2C9 and CYP3A4; in addition to having acute and subchronic toxic potential. Therefore, muricatacin presented different characteristics with greater bioavailability and less acute toxicity. Docking showed acetogenins perform hydrophobic interactions, hydrogen bonds and covalent bonds to maintain both affinity and stability between caspase 3 and the selected ligands, and the cis-uvariamicin IV was the most promising compound for this parameter. Furthermore, the literature has shown the cytotoxic activity of acetogenins. The chloroform fraction from methanolic extract of *Annona muricata* seeds presented activity against triple-negative breast cancer, both *in vitro* and *in vivo*, which would be associated with the presence of acetogenins, mainly muricatacin. However, muricatacin and cis-uvariamycin IV would probably be the most promising molecules in the present study, with cis-uvariamycin IV being safer as a possible adjuvant for antitumor drugs.

## Data Availability

The raw data supporting the conclusion of this article will be made available by the authors, without undue reservation.
